# Gaz Alafi: A Traditional Dessert in the Middle East With Anticancer, Immunomodulatory, and Antimicrobial Activities

**DOI:** 10.3389/fnut.2022.900506

**Published:** 2022-07-01

**Authors:** Meena A. Al Safi, Hasan M. Rashid, Fatma U. Afifi, Wamidh H. Talib

**Affiliations:** ^1^Department of Clinical Pharmacy and Therapeutics, Applied Science Private University, Amman, Jordan; ^2^Department of Pharmaceutical Chemistry and Pharmacognosy, Applied Science Private University, Amman, Jordan

**Keywords:** antiproliferation, antimicrobial, immunomodulatory, functional food, *in vivo*

## Abstract

**Background:**

From the earliest times, manna has been widely used as a tasty local sweet or folk medicine. The type of manna being investigated in the present study is called Gaz-alafi, a mixture of insect and *Quercus brantii* leaves secretions from oak forests in the north of Iraq and west of Iran.

**Methods:**

Aqueous and ethanol extracts were prepared as decoction. Various phytochemical tests were conducted to analyze manna composition, including total phenolic contents using the Folin-Ciocalteu method and LC-MS. Gallic acid and catechin were detected in both extracts, in addition to tiliroside presence in ethanol extract, which added more value to the phenolic content of ethanol extract. Cytotoxic activities of Gaz alafi were evaluated against breast cancer cell lines and compared to normal cell lines and doxorubicin using the MTT assay. Antimicrobial properties were assessed against *Escherichia coli, Pseudomonas aeruginosa, Staphylococcus aureus, Bacillus subtilis*, and *Candida albicans* using the dilution method of the micro-titer plate. Serum levels of IFN-γ, interleukin-2 (IL-2), interleukin-4 (IL-4), and interleukin-10 (IL-10) were measured using ELISA. The effect of extracts on splenocyte proliferation was evaluated using the lymphocytes proliferation assay. Macrophage function was evaluated using the nitro blue tetrazolium assay, whereas pinocytosis was evaluated using the neutral red uptake assay. Ten days after tumor inoculation, changes in tumor size, survival rates, levels of alanine aminotransferase (ALT), aspartate aminotransferase (AST), and creatinine were measured.

**Results:**

The growth of cancer cells was inhibited by Gaz alafi ethanol extract. An alteration in IFN- γ, IL-2, and IL-4 levels toward antiproliferation immune response were reported for both extracts. The aqueous extract efficiently stimulated lymphocyte proliferation, phagocytosis, and pinocytosis, followed by the ethanol extracts with moderate activity. After treating the mice with ethanol extracts, a significant reduction in tumor size and several undetected tumors were recorded.

**Conclusions:**

Gaz alafi extracts (aqueous and ethanol) are promising sources for anticancer and immunostimulatory agents. Further studies are needed to fully identify the chemical composition of Gaz alafi extracts.

## Introduction

Cancer is considered as one of the most serious public health problems worldwide. Its progression and mortality quickly grow, causing more than eight million deaths annually (1, 2). Female breast cancer has exceeded lung cancer as the most diagnosed cancer, with an estimated 2.3 million new cases (11.7% of total cases). In fact, it has been the leading cause of death for females in present days (3, 4).

Nowadays, biological molecules, drugs, and immune-mediated therapies are being used to treat cancer. Until now, the scientists has not reached the excepted therapy level that reduces the mortality rate and decreases the prolonged survival time for metastatic disease (1). The side effects and toxicity are significant drawbacks in conventional radiotherapy and chemotherapy, encouraging scientists to find alternative cancer therapies to enhance the efficiency of current treatments and reduce toxicity and side effects (5).

Natural products are considered as an attractive source for alternative anticancer therapies, which could play a crucial role in the treatment of commonly occurring cancers worldwide by targeting proliferating tumor cells. The therapeutic effects of these products could be mediated by multiple pathways, such as indirect effects on the immune systems by increasing (immunostimulatory) or decreasing (immunosuppressive) the immune response (6). Another way to target the cancer is by affecting one of its causes, such as the free radicals responsible for various ailments such as cell aging and are considered a triggering factor in cancer (7).

World Health Organization (WHO) estimated that 80% of world inhabitants depend on plant-derived traditional medicines in health care, and, in 2050, the global market of natural products is expected to grow and reach $5 trillion (8). Phytochemicals used as chemo prevention are of great interest and are considered inexpensive, acceptable, readily applicable, and accessible for cancer control and management. Natural products offer protective and therapeutic actions with relatively low toxicity compared to synthetic anti-cancer drugs, which cause nonspecific killing of cells. Currently, many phytochemicals are in the preclinical or clinical trials for cancer chemoprevention. These natural products could be found in vegetables, fruits, plant extracts, and herbs. Although their mechanism of action is unclear, the consumption of fruits and vegetables reduces carcinogenesis in a wide variety of types of cancers (9, 10). In addition to the anticancer effects of the natural products, they are considered as an essential ingredient of functional foods and dietary supplements that can support the body systems against various diseases, such as bacterial infections, cardiovascular, gastrointestinal, and inflammatory conditions (11).

For centuries, in the north of Iraq and Iran, a popular sweetmeat has been made with a substance collected from the plants. The plant produces the exudate in response to an attack of insects. The sweet exudate that results from this infection is commonly referred to as *gaz*. manna (Gaz alafi). It is known from the earliest times and is widely used as a tasty local sweet or, in folk medicine, as laxatives. It is also used in the traditional medicine of Iran as a sedative, antipyretic, and analgesic and as a treatment of chickenpox, rubella, and related itching (12). The same study showed that the Gaz alafi contains the highest concentration of iron and minerals, essential elements for human well-being, compared with other types of manna. They found that it can be used by those who have ulcerative colitis, bleeding disorders, leukemia, immune system disruption, or by those who suffered from blood loss. Taking manna in a diet could benefit persons with low white blood cells or those with lowered immunity conditions. Also, it can provide energy for growth and improve the quality of life (12–14).

There is more than one source of manna. It could be produced naturally from plants without any apparent stimulus or artificially by human-made wounds, used in the commercial production of manna sweets. Another source is the animal origin and is only produced indirectly from plants, although often confused with direct exudations from the plants themselves (15, 16). It is a stiff, resinous, sweet natural product that appears on the leaves of some *Quercus* species, including (*Q. brantii* Lindi.) from the family Fagaceae. In this study, the *Q. brantii* dry leaves with manna exudate was used. The excretion on the leaves was obtained from two insects known as (*Thelaxes suberi* Del.) and (*Tuberculoides annulatus* Hart) from the Aphidoidea family.

To the best of our knowledge, no research has been done demonstrating manna's anticancer and immunomodulatory effects. The present study will investigate these activities using different extracts prepared from Gaz alafi obtained from *Q. brantii*. In addition to the *in vitro* and *in vivo* experiments, chemical analysis of the manna extracts and antimicrobial evaluation were tested.

## Materials and Methods

### Plant Samples and Preparation

One kilogram of Gaz alafi (as a solid mass of *Q. brantii* leaves with manna exudate) was purchased from an Iraqi farmer in the Penjwen district of Sulaymaniyah (north of Iraq) in September 2020. The dry leaves of *Q. brantii* contained manna exudate. Since the origin of this material was Sulaymaniyah, this sample will be referred to throughout the research, with the local name “Gaz alafi” indicated that the studied material is not purely the leaves of *Q. brantii*. The species were identified by Mr. Nizar Obaidat (Ministry of Agriculture, Jordan). Images of the manna and the plant are found in the Supplementary Figure 1.

Two different extracts were prepared with distilled water (DW) and 70% ethanol using the ratio of plant to solvents at 1:10 w/v by boiling the samples separately for 3–5 min. Extracts were kept on the bench overnight at room temperature (RT) and then filtered. Ethanol extract was concentrated using a rotary evaporator, and the aqueous extract was dried completely using a lyophilizer, and then both extracts were kept at −20°C until used (17). Upon the extraction of 100 g, 70% ethanol extracts were concentrated to yield 58 g (58%) dry material, while aqueous extract yielded 4 g (4%) only.

### Total Phenolic Content

The total phenolic content (TPC) in Gaz alafi extracts was determined according to the Folin-Ciocalteu (F-C) procedure described in the literature (18). A 200 μL of 1 mg/ml from each extract was diluted with 10-ml distilled water (DW) in 25 volumetric flasks, and then 200 μL of Folin-Ciocalteu reagent was added. After 5 min, 800 μL of 20% sodium carbonate was added, and the volume was adjusted to 5 ml with DW. The resulting greenish-blue solution was incubated at room temperature for 1 h in a dark place. The absorbance was measured at 750 nm. The absorbance values were then read from the standard curve obtained from the same procedure on the gallic acid standard (Supplementary Figure 6) (19). Results were represented as mg GAE/g dry weight of extracts.

### Liquid Chromatography-Mass Spectrometry

A Bruker Daltonik Impact II ESI-Q-TOF System with a Bruker Daltonik Elute UPLC system (Bremen, Germany) was used to screen the compounds of interest in the extracts. The extract samples were dissolved in 2 ml DMSO and made up to 50 ml using acetonitrile. Then, the samples were centrifuged at 4,000 rpm for 2 min and transferred to autosampler where 3 μL was injected (59 standards were used to identify ms/z and the retention time).

After chromatographic separation, high-resolution Bruker TOF MS was utilized to determine m/z and the exact retention duration of each analyte. The Ion Source Apollo II ion Funnel electrospray source was used to power this instrument. The capillary voltage was 2,500 V, the nebulizer gas pressure was 2 bar, the dry gas (nitrogen) flow rate was 8 L/min, and the dry temperature was 200°C. The mass resolution was 50,000 FSR, and the mass accuracy was one ppm (Full Sensitivity Resolution). The repetition rate of the TOF was up to 20 kHz. Chromatographic separation was achieved using an Elute UHPLC connected to a Bruker Impact II QTOFMS.

### Antiproliferation Study

#### Animals

This research was carried out following accepted ethical standards. The Research and Ethical Committee approved all experimental protocols at the Faculty of Pharmacy, Applied Science Private University (Approval No: 2015-PHA-05). A total of 54 healthy female Balb/C mice, weighing between 21 and 25 grams and aged 6 to 8 weeks, were used in this study. The mice were housed in well-ventilated rooms with room temperature (25°C), 50–60% humidity, and alternate dark and light cycles every 12 h. They were kept in cages with bedding made of wood shavings, a special water bottle, and food.

#### Cell Lines and Cell Culture Conditions

Four different breast cancer cell lines (T47D, MCF-7, MDA-MB231, and EMT-6) and Human Gingival Fibroblast (HGF) were used in this study. The cells were cultured using suitable tissue culture media supplemented with L-glutamine, serum, and antibiotics. A humidified atmosphere of 5% CO_2_ at 37°C was applied to incubate different cell lines (20). T47D and MCF-7 cell lines were cultured in a complete RPMI 1,640 medium. The EMT6 cell line was cultured in complete Minimum Essential Medium (MEM), while Dulbecco's Modified Eagle Medium (DMEM) was used for the MDA-MB231 cell line.

#### Cytotoxicity and Antiproliferative Activity Assay

Cell viability was measured using MTT [3-(4,5-Dimethylthiazol-2-yl)-2,5-diphenyltetrazolium bromide)] assay kit (Bioworld, UK). The cells were dispensed (100 μL/well) into each well of 96-well tissue culture plates at a density of 15,000 cells/well. After a 24-h incubation period, the medium in each well was removed, and the cells were treated in triplicate with varying doses of the extracts (initially dissolved in DMSO with final concentration not exceeding 1%). Doxorubicin (5–0.015 mg/ml) was used as a positive control. After 48-h incubation, media were withdrawn from each well, rinsed with phosphate buffer saline (PBS), and replaced with fresh media, followed by adding 20 μL of thiazolyl blue tetrazolium bromide solution, with a 3-h incubation period. One hundred microliters of DMSO was added to each well to terminate the reaction. The cells treated with 1 DMSO were used as negative controls. Microsoft Excel software was used to apply further calculations that estimate the percentage of survival cells and calculate the IC50 values (20), where OD is optical density:


$$\left(Percentage\;of\;cell\;viability\;(\%)\;=\frac{\left(OD\;of\;treated\;cell\right)}{\left(OD\;of\;control\;cell\right)}\times 100\right)$$

#### Acute Toxicity Test of Gaz Alafi Ethanol Extract

A pilot study was conducted on a small group of mice to select the dose ranges for actual LD_50_ (median lethal dose). Gaz alafi ethanol extracts were dissolved in PBS and 5% tween 20. Four female mice (6 weeks old, 20–23-g weight) were injected intraperitoneally (IP), with a plant extract dose (2 g/kg), which was obtained from the literature (21). The mice were observed for 24 h for any mortality. The next-day dose was adjusted by increasing 1.5 times if tolerated or decreasing 75 times if it showed toxicity. The maximum non-lethal and minimum lethal doses were used as lower and upper limits to calculate LD_50_ doses. After the pilot study, five groups (n = 6) of mice were injected IP with concentrations of (1.7, 3, 4.5, 6.5, and 10 g/kg). The sixth group was used as a negative control and was injected with PBS. The mice were monitored for 24 h for mortality and general behavior. The concentration that causes 50% mortality was recorded as LD_50_. The actual LD_50_ was determined using the arithmetical method of Karber (22).

#### Antitumor Effect of Gaz Alafi in Mice Model Experiment

Subcutaneous injection was used to inoculate 100 μL (150,000 cells) of EMT-6/P cells in the abdominal area of female Balb/C mice. Ten days after tumor inoculation, the mice were subjected to daily intraperitoneal (IP) injections with 6 mg/kg/day ethanol extract for 10 days (Table 1). After 10 days of treatment, tumor volumes were measured using a digital caliper to define the length and width. Tumor volumes were calculated using the following equation (23):


$$V=\frac{L\times W^{2}}{2}$$

**Table 1:** *In vivo* experiment intraperitoneally (IP) injection of treatment (ethanol extract) and control groups.

**Groups**	**Injection**
Treatment	6 mg/kg/day
Control	100 μL Tween 20 and 100 μL (PBS) daily

where: V, L, and W are the volume, length, and width of the tumor, respectively.

#### Evaluation of Liver and Kidney Function in Treated Mice

Aspartate transaminase (AST), alanine transaminase (ALT), and creatinine in serum samples were measured using commercially available kits, following kit instructions (BioSystems, Barcelona, Spain). Serum levels of liver enzymes AST and ALT were investigated for Gaz alafi, the treated mice, the untreated mice, and the normal mice without tumor. Serum samples were collected, and the reagent was mixed according to the protocols to prepare working reagents. The working reagents were incubated at 37°C, which is the optimal reaction temperature. In cuvette, 50 μL of each sample was mixed with 1 ml of working reagent, incubated for 1 min, and the initial absorbance was recorded. Absorbance readings were recorded also after 0, 1, 2, and 3 min. The spectrophotometer was set to read absorbance at 340 nm. Creatinine serum levels were investigated for the same groups. The reagents were mixed and incubated at 37°C. In a cuvette, 100 μL of each sample was mixed with 1 ml of working reagent, Absorbance readings were recorded after 30 s and 90 s. The spectrophotometer was set to read absorbance at 500 nm. Working reagents in all the tests were used as blanks.

### Immunomodulatory Assay

#### Preparation of Murine Splenocytes

Balb/C mice were sacrificed, and the spleens were removed aseptically. The spleen cells were passed through the mesh of a tissue grinder. The cell suspension was washed for 10 min using RPMI-1640 media and then resuspended in 1 mol/L NH_4_Cl to eliminate red blood cells (RBC). The cells were centrifuged after 10 min and resuspended in RPMI-1640 media. After that, splenocytes were cleansed, counted, and used in various tests (24).

#### Determination of Cytokines Levels in Activated Lymphocytes

The serum levels of IFN-γ, IL-2, IL-4, and IL-10 were measured using a Mouse T helper Th1/Th2 ELISA kit (Invitrogen by theme fisher scientific, Australia). Serum samples from treatment and control groups were prepared from mice blood samples. Measuring cytokine levels was based on the kit instructions. Standard curves for the tests are found in the Supplementary Figures S2–S5.

#### Lymphocyte's Proliferation Assay

Splenocytes suspension was made (2 × 10^6^ cell/ml) in RPMI-1640 media (supplemented with 50-U/ml penicillin, 50-U/ml streptomycins, and 10% FBS), and then it was seeded into a 96-well culture plate, containing either 5 μg/ml Con A (Concanavalin A) or 4-μg/ml LPS (lipopolysaccharide). Then, 100 μL of (5–20 mg/ml) of the extracts was added (in triplicate). The plate was incubated for 48 h under 5% CO_2_ and a humidified atmosphere of 95% air at 37°C temperature. Later, 10 μL of (5 mg/ml) MTT [3-(4, 5-Dimethylthiazol-2-yl)-2, 5- diphenyltetrazolium bromide] solution from an assay kit (Bioworld, UK) was added to each well and incubated for 4 h, followed by the addition of 100-μL DMSO. The absorbance was measured at 550 nm using an ELISA microplate reader. Results were expressed as a percentage of proliferation (%) compared to the control cells. The same procedure was repeated without Con A and LPS (25, 26).

#### Macrophage Isolation

Peritoneal macrophages (PEM) were isolated from peritoneal mice cavities. The cervical dislocation method was applied to euthanize the mice, followed by abdominal cavities exposure, dispensing 5-ml ice-cold PBS, and gentle massaging, and then fluid was withdrawn. This method was performed five times. After centrifuging, pellets were suspended in an RPMI 1640 medium, supplemented with 10% fetal bovine serum (FBS) and penicillin-streptomycin (100 U/ml) (all chemicals were supplied from Sigma, Chennai) and let to adhere for 3 h at 37°C in an 5% CO_2_-humidified incubator. These cells were used in the tests outlined below (27).

#### Phagocytic Activity Assay

The nitro blue tetrazolium (NBT) reduction assay was carried out according to the method of Rainard (28). PEM (5 x 10^6^ cells/well) was cultured with (5–20 mg/ml) extracts concentrations for 48 h at 37 °C. Next, 20 μL yeast suspension (5 × 10^6^ cells/ml in PBS) and 20 μL NBT (1.5 mg/ml in PBS) were added to the wells. The control wells contained 20 μL PBS and 20 μL DMSO, followed by incubation for 60 min at 37 °C, air-drying, and addition of 120 μL (2-M KOH) and 140 μL (DMSO). The absorbance was measured for the turquoise blue solution at 570 nm using the plate reader. The measurement of NBT reduction was estimated as below (25, 29), where OD is (optical density):


$$Phagocytic\;index\;=\frac{\left(OD\;sample\;-\;OD\;control\right)}{\left(OD\;control\right)}\times 100$$

#### Pinocytic Activity Assay

The neutral red uptake method was used to determine the effect on macrophage function. Peritoneal mice macrophages were collected and cultured for 48 h with extracts concentrations of (5–20 mg/ml), employing a 96-well plate, followed by the addition of 100 μL of neutral red solution (7.5 mg/L in PBS), incubation for 2 h, and washing with PBS. Then, 100 μL of cell lysis solution (ethanol plus 0.01% acetic acid, ratio of 1:1) were added to each well. Afterward, the cells were incubated overnight. The optical density was determined at 540 nm. Absolute OD values representing dye uptake have been used to measure pinocytic activity (24, 25).

### Antibacterial Assay

The antibacterial and antifungal potential was studied using the Gaz alafi extracts dissolved in DMSO (dimethyl sulphoxide). The bacterial strains used in this study were gram-negative bacteria, including *Escherichia coli* (ATCC^®^25922™), *Pseudomonas aeruginosa* (ATCC^®^27853™), gram-positive bacteria, including *Bacillus subtilis* (ATCC^®^6633™), *Staphylococcus aureus* (ATCC^®^6538™), and the yeast *Candida albicans* (ATCC^®^90028™). The dilution method of the micro-titer plate was used to determine the minimal inhibitory concentration (MIC). Each extract (100 mg) was dissolved in 40-μL DMSO, and Muller Hinton broth (MHB) was added up to l ml. Positive controls of gentamycin and amphotericin B (for fungus) were prepared at concentrations of (7.8–1,000 μg/ml). The plates were inoculated with bacterial suspension (100 μL/well) and incubated at 37°C for 24 h. Then, the turbidity was measured using an ELISA absorbance microplate reader at 620 nm for bacteria and 530 nm for the fungus. The MIC was determined as the least concentration required to microbial bacterial growth (30–32).

### Statistical Analysis

Using the SPSS statistical package version (version 21), data in this study were presented as the mean ± SEM (standard error of mean) of independent trials. SPSS one-way ANOVA was used to establish the statistical significance between the groups. IC_50_ estimates were determined using non-linear regression analysis. P-value of (< 0.05) was considered significant.

## Results

### Determination of Total Phenolic Contents

**Figure 1:**
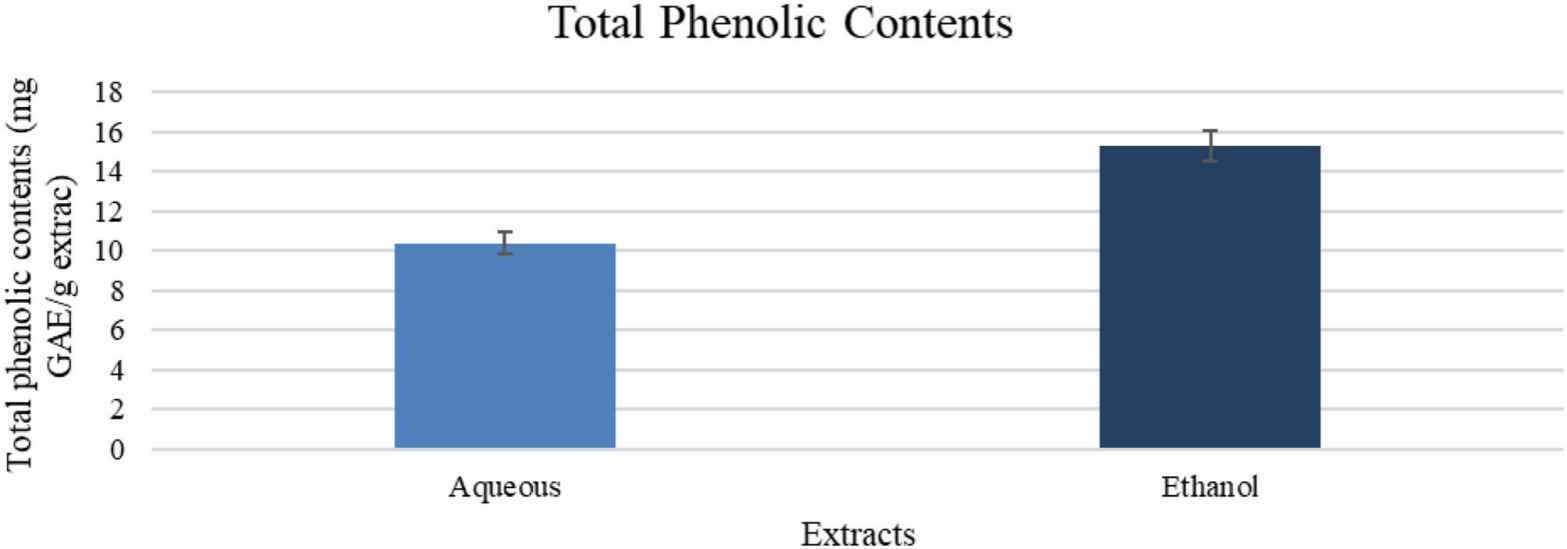
Total phenolic contents (mg GAE/g of extract). Results are expressed as means (bars) ± SEM (lines), *n* = 3.

Aqueous and ethanol extracts showed the phenolics content equivalent to gallic acid, with a value of 10.4 and 15.3 mg GAE/g extract, respectively, as shown in Figure 1.

### Liquid Chromatography-Mass Spectrometry

Qualitative analysis of the aqueous extract using LC-MS compared to the standards available for this study (see Supplementary Figure S9) revealed the presence of two compounds in the aqueous extract, including high amount of gallic acid (65.2%), followed by catechin (34.79%). Both compounds were detected in the ethanol extract with a percentage of 58.03 and 15.79%. In addition to a third compound, tiliroside (26.19%), which is a glycosidic flavonoid. These results are semiquantitative and showed the percentage among the detected compounds. The three detected compounds were further analyzed to calculate their concentration in the extract (Table 2). Other compounds were below the limit of quantitation (LOQ) value, and these cannot be quantified. The detected compounds are shown in Table 2. The detailed LC-MS chromatograms were found in Supplementary Figures S7, S8.

**Table 2:** The components of Gaz alafi extracts using LC-MS qualitative system.

**Name**	**Molecular Formula**	**Molecular Weight**	**Retention Time [min]**	**Amount (Aqueous)**	**Amount (Ethanol)**	**% Of the Identified Compounds (Aqueous)**	**% Of the Identified Compounds (Ethanol)**
Gallic Acid	C_7_H_6_O_5_	170.02	1.11	0.0344 mg/kg	0.024 mg/kg	65.2	58.03
Catechin	C_15_H_14_O_6_	290.08	2.81	0.0258 mg/kg	0.0354 mg/kg	34.79	15.79
Tiliroside	C_30_H_26_O_13_	594.14	9	-	0.0035 mg/kg	-	26.19

### *In vitro* Antiproliferative Assay

**Figure 2:**
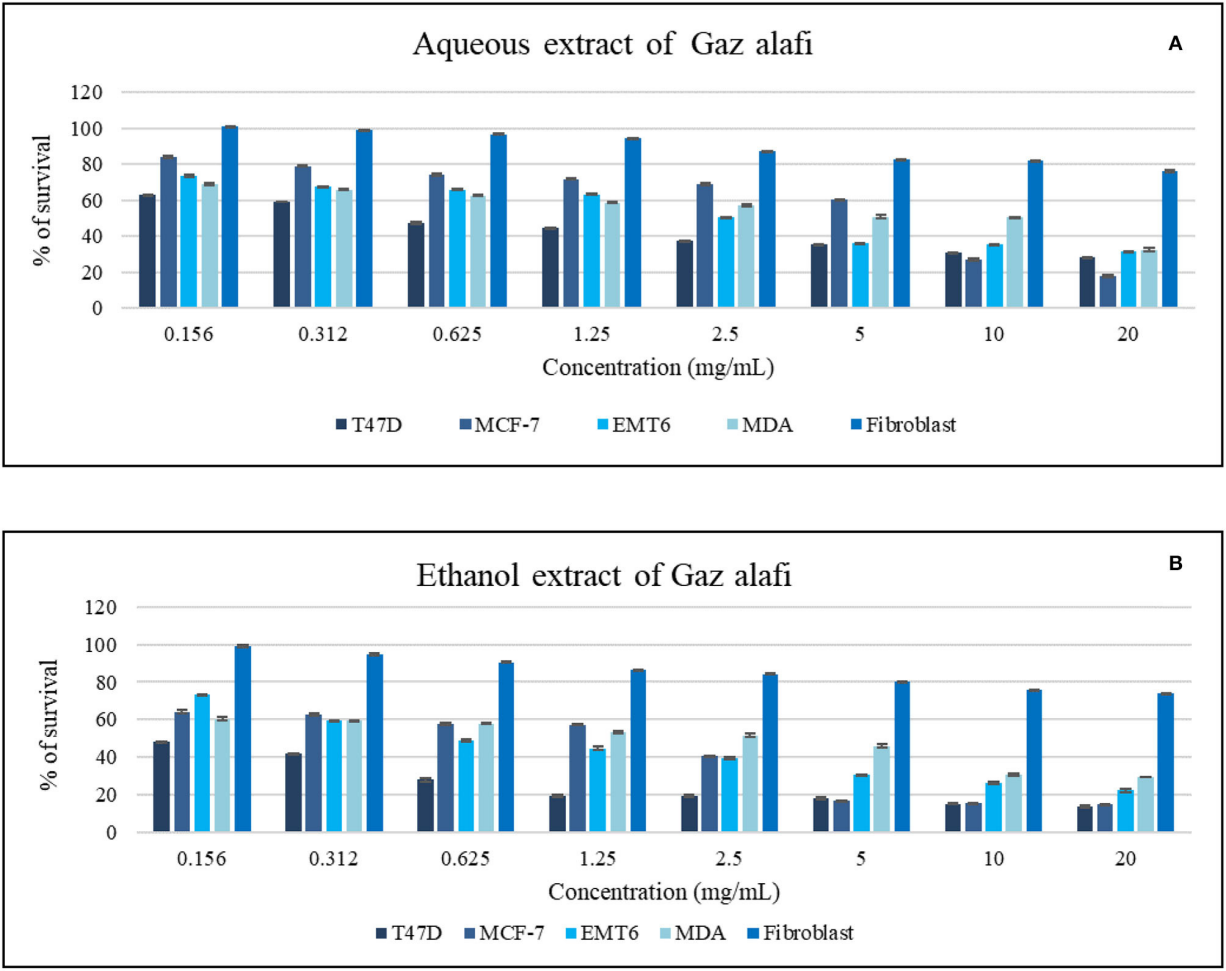
Antiproliferative activity of aqueous **(A)** and ethanol **(B)** extracts of Gaz alafi on breast cancer and fibroblast cell lines using a concentration range of (0.156-20 mg/ml). Results were represented as mean (bars) ± SEM (lines), (*n* = 3).

The effects of Gaz alafi extracts on the proliferation of several breast cancer cell lines and fibroblast normal cell lines are shown in Table 3. Both aqueous and ethanolic extracts exhibited the cytotoxicity against T47D cells, with IC_50_ values of 0.80 and <0.15 mg/ml, respectively. However, higher activity was observed for the ethanolic extract on the other investigated cell lines (MCF-7, MDA-MB231, EMT-6) (Figure 2). Regarding normal cell lines (fibroblast), no toxicity was observed for Gaz alafi extracts. Results from literature showed IC_50_ values of doxorubicin against MCF-7 were 0.68 ± 0.04 μg/ml (33), 3.16 μM for MDA-MB231, and 8.53 μM for T47D (34).

**Table 3:** The effects of Gaz alafi extracts on the proliferation of breast cancer cell lines and Fibroblast normal cell line represented as IC_50_ (mg/ml) as mean ± SEM, *n* = 3.

**Extracts**	**T47D**	**MCF-7**	**EMT-6**	**MDA-MB231**	**Fibroblast**
Aqueous	0.80 ± 0.08	6.07 ± 0.40	2.5 ± 0.01	4.90 ± 0.05	>20
Ethanol	<0.15	1.36 ± 0.02	1.16 ± 0.18	3.02 ± 0.09	>20
Doxorubcin	0.00032 ± 0.04	0.005 ± 0.40	0.00057 ± 0.22	0.00082 ± 0.03	>0.2

Various extract and doxorubicin concentrations were tested. The activity was noticed against T47D cells at the lowest concentration (0.156 mg/ml). In contrast, in the EMT6 cell line case, the effect started around 0.625 mg/ml. On the other hand, higher concentrations of aqueous extract were needed to have antiproliferative activity as shown in Figure 2.

### Immunomodulatory Assay

#### The Evaluation of Cytokines Levels

At a dose of 20 mg/ml, treatment with ethanol extract caused IFN-γ, IL-2, and IL-10 serum levels to decrease to 13, 16, and 3 pg/ml, respectively. The same treatment caused IL-2 serum levels boosting (578 pg/ml) compared with untreated tumor-bearing mice that exhibited values of 185, 107, 30, and 52 pg/ml for IL-2, IL-4, IL-10, and IFN-γ, respectively.

**Figure 3:**
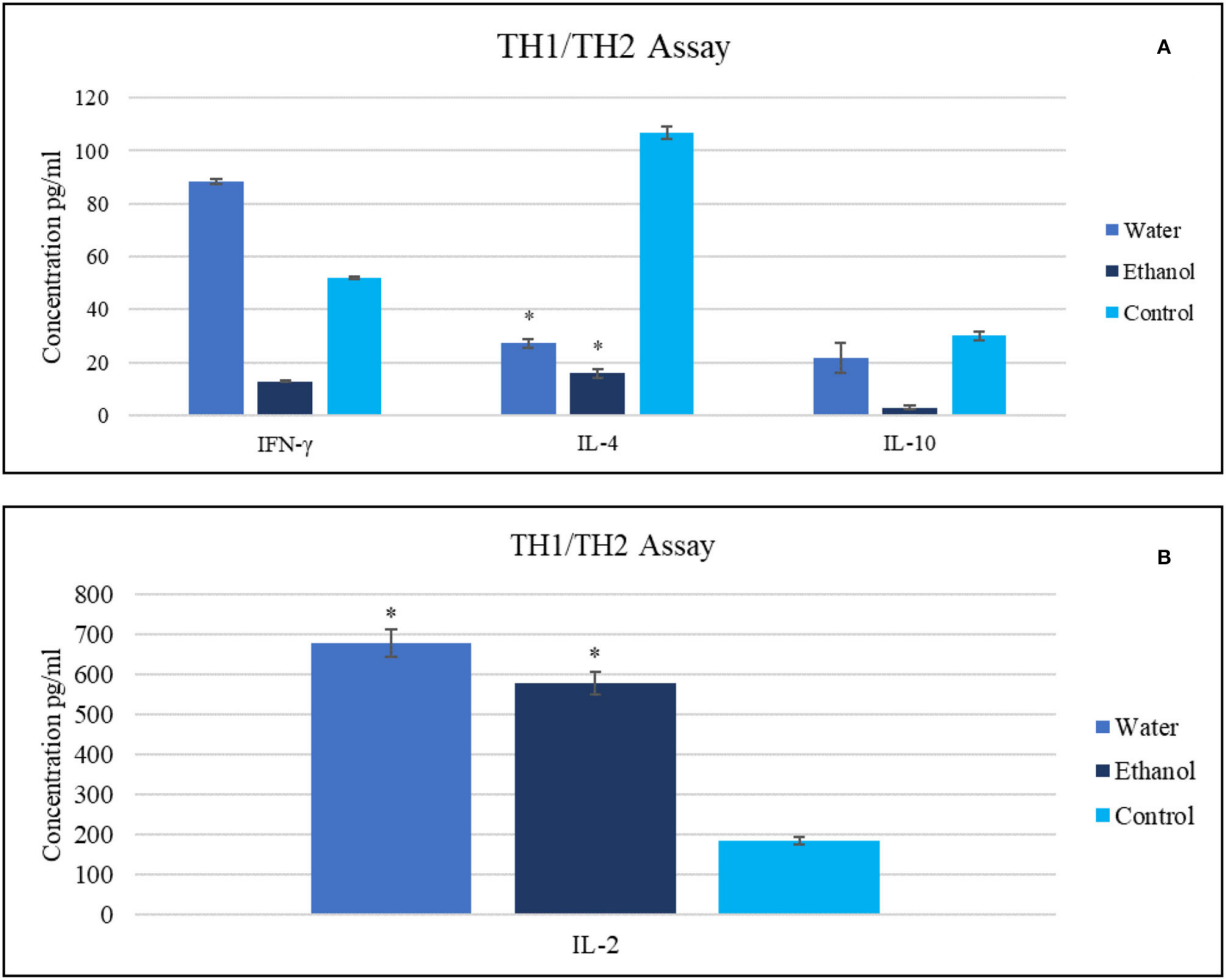
The impact of Gaz alafi extracts (20 mg/ml) on **(A)** IFN- γ, IL-10, IL-4, and **(B)** IL-2 levels. Each treatment was performed in duplicate. The highest levels of IFN-γ and IL-2 were detected after aqueous therapy. IFN, interferon; IL, interleukin. ^*^P < 0.05 (statistically significant).

In contrast, different results were obtained for mice treated with aqueous extract, with an increase in IFN-γ and IL-2 serum levels to 88 and 680 pg/ml, respectively. This treatment also decreased the IL-4 level (27 pg/ml) and reduced the IL-10 level (22 pg/ml) (Figure 3).

#### Lymphocyte's Proliferation Assays

**Figure 4:**
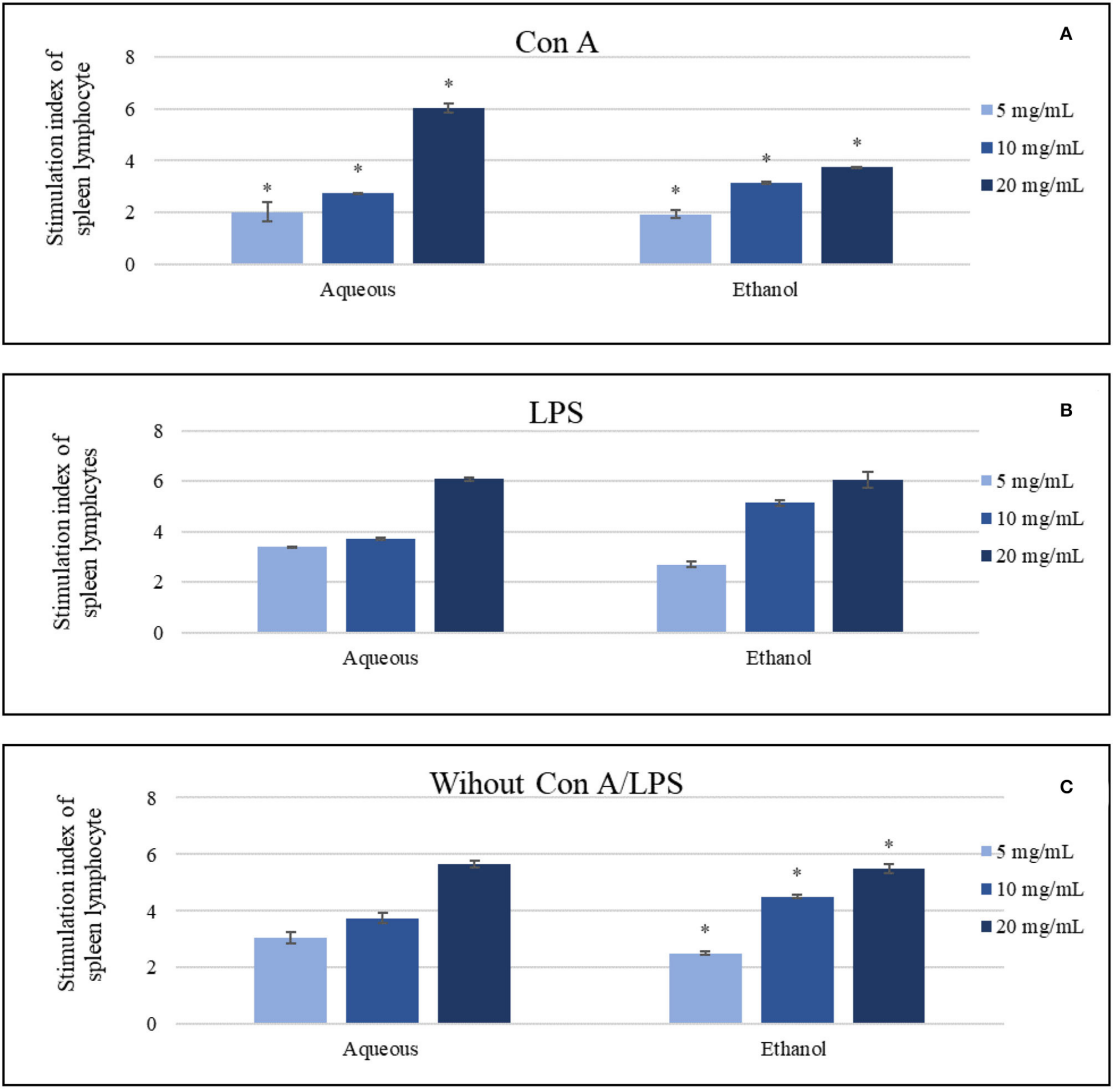
The impact of the aqueous and ethanol extracts (5-20 mg/ml) on the splenic lymphocytes in comparing to negative control, which was 1, **(A)** in the presence of concanavalin A, **(B)** in the presence of lipopolysaccharide, **(C)** in the absence of both Con A and LPS. Results were represented as mean (bars) ± SEM (lines), ^*^P < 0.05 (statistically significant).

Aqueous extract was the most effective extract at a concentration of (20 mg/ml) compared to the control (*p* < 0.05) in the presence and absence of mitogens. Ethanol extract revealed active but slightly lower results than the aqueous extract. The stimulation index of aqueous extract (20 mg/ml) was around 6.04 and 6.08 in Con A and LPS-stimulated cells, respectively, where ethanol showed 3.74 and 6.06 at the same tests. In contrast, the same indexes of aqueous and ethanol extracts (20 mg/ml) in the absence of mitogens were 5.65 and 5.5, respectively (Figure 4). Other treating concentrations exhibited various effects.

#### Phagocytosis Assay

**Figure 5:**
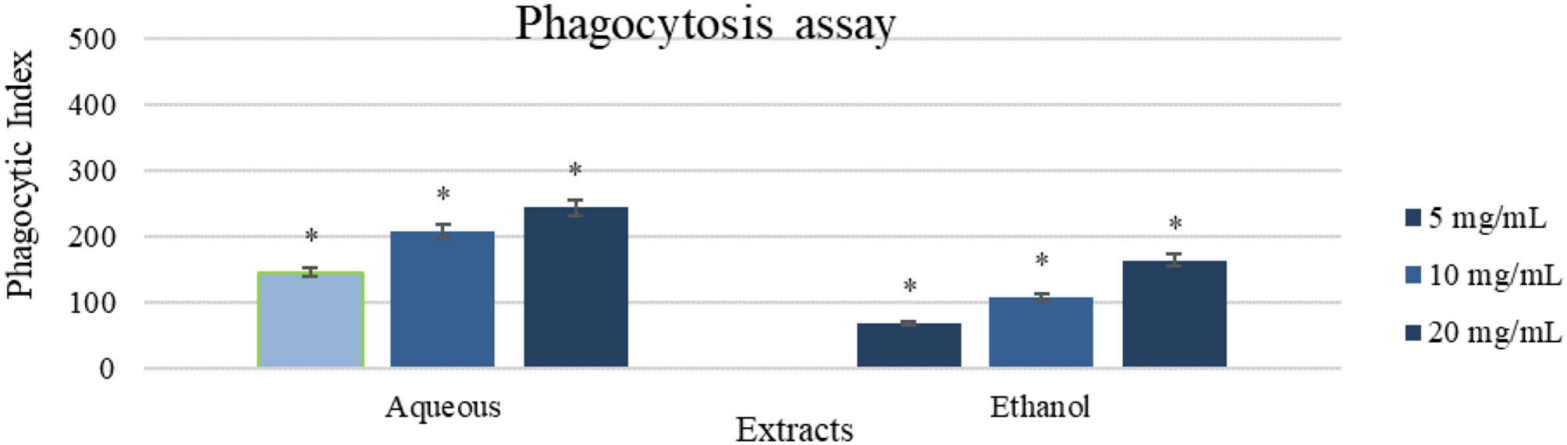
A phagocytic assay of peritoneal macrophage treated with the aqueous and ethanol extracts (5-20 mg/ml). Results were represented as mean (bars) ± SEM (lines), (*n* = 3). ^*^P < 0.05 (statistically significant).

The aqueous extract (20 mg/ml) revealed the greatest inducement of peritoneal phagocytic activity (Phagocytic index of 243) compared to the control (which was zero), followed by the ethanol extract (20 mg/ml) (Phagocytic index of 164). Treated cells exhibited a concentration-dependent behavior as shown in Figure 5.

#### Pinocytosis Assay

**Figure 6:**
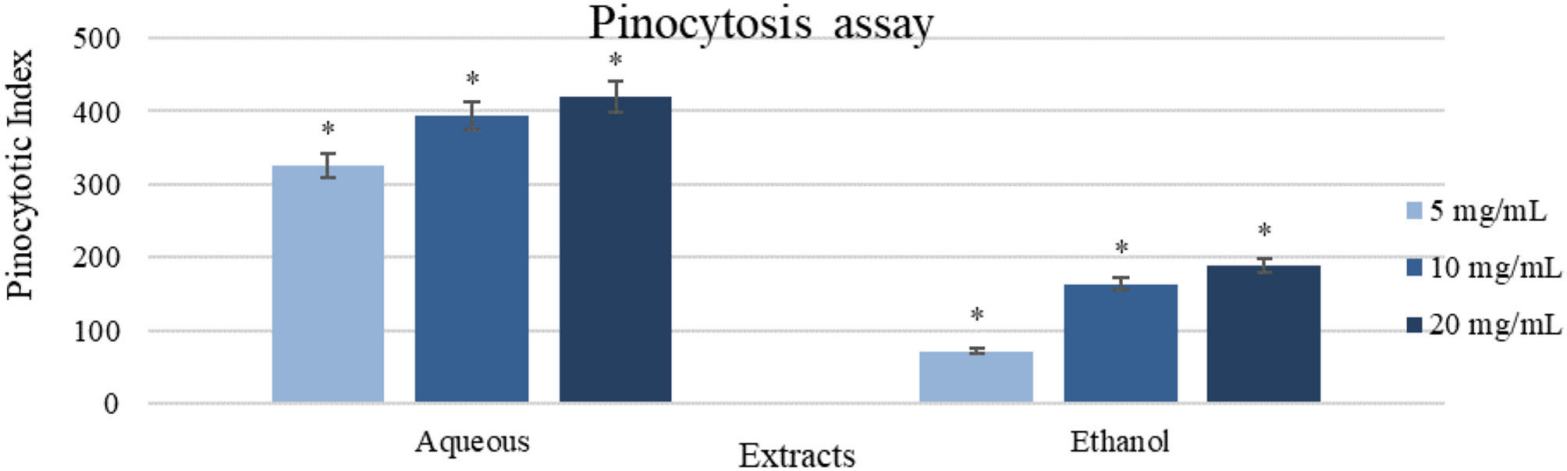
A pinocytosis assay using the neutral red method on peritoneal macrophages treated with different concentrations of the extracts (5-20 mg/ml). Results were represented as mean (bars) ± SEM (lines), (*n* = 3). ^*^P < 0.05 (statistically significant).

Aqueous extract (20 mg/ml) showed a high impact with pinocytic index value of (421.75 ± 21.09) as shown in Figure 6. Oppositely, ethanol extract (20 mg/ml) was less active in comparison with the aqueous extract (187.5 ± 9.38), and the result of the control was zero.

### *In vivo* Antiproliferative Assay

#### Acute Toxicity Test (LD_50_ Determination)

A small group of mice was used in a pilot experiment to determine the actual LD_50_ (median lethal dose). No toxicity and no mice death were found, starting from 2g/kg dose, reaching 10 g/kg using the Karber technique (22). The treatment dose (6 g/kg) was decided based on the extract solubility.

#### Antitumor Effects on EMT6/P Cells Implanted in Mice

**Figure 7:**
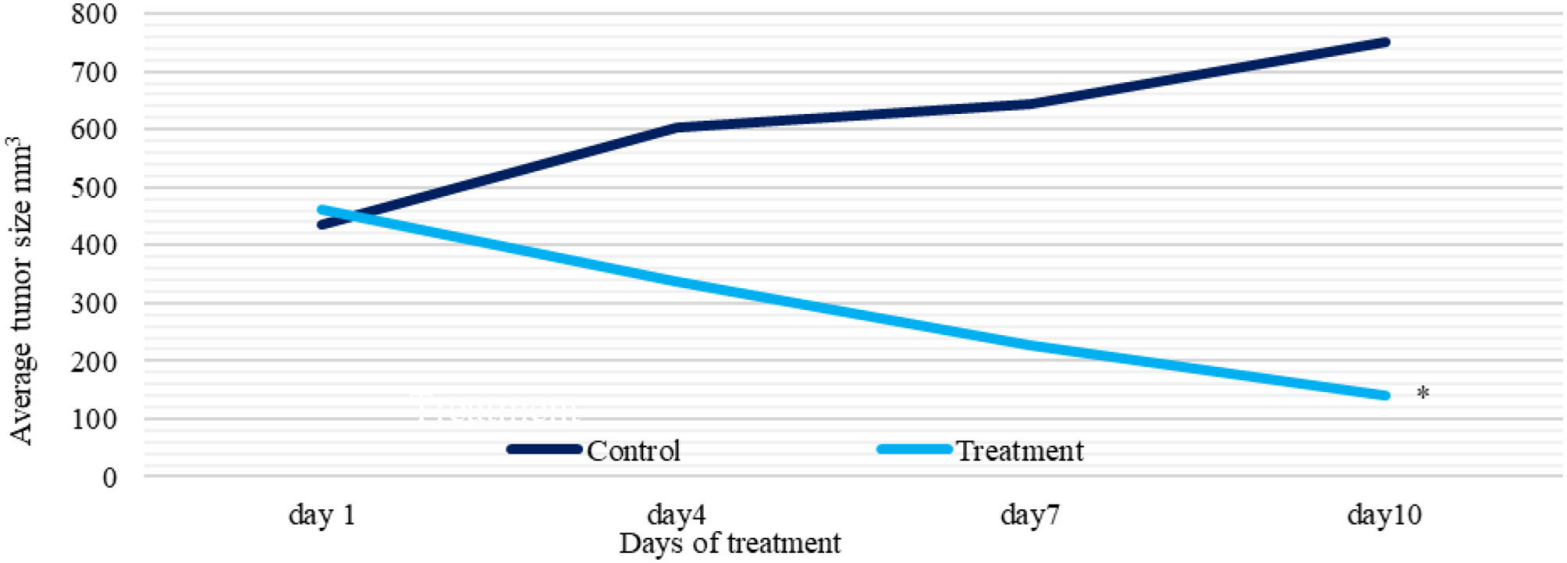
A plot of change in average tumor size (mm 3) vs. time (days) of treatment and control groups. Tumor sizes were measured with 3-day intervals. ^*^P < 0.05 (statistically significant).

**Figure 8:**
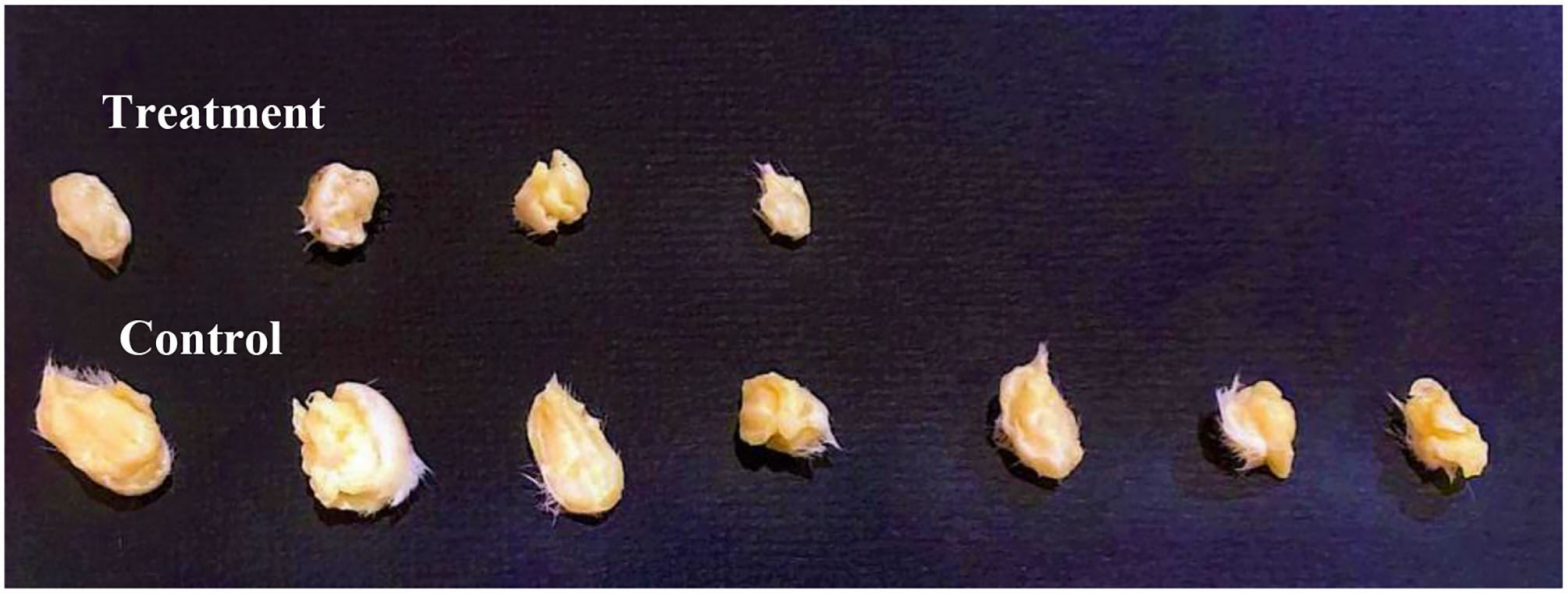
Comparison between group tumor sizes at Day 10, *N* = 7. Ethanol extract treatment resulted in lower tumor sizes and three undetected tumors compared to the control group.

Tumors were inoculated in Balb/C females; after 10 days, tumor sizes were measured. Ethanol extract was injected intraperitoneally (IP). The treatment group received 6 g/kg/day. The control group represented the vehicle-treated mice with tumor. Observations revealed a significant reduction in the treatment group tumor sizes (70%) (*p* = 0.003). While, in the control, tumor growth was increased by (72%), and one mouse died. The treatment group caused undetected tumors by (43%), with no recorded deaths (Figures 7, 8 and Table 4).

**Table 4:** Effect of Gaz alafi ethanol extract treatment on tumor size, weight, tumors detection, and deaths percentages, in the EMT-6/P cell line, where (mm3) is a cubic millimeter (*n* = 7).

**Groups**	**Av. Initial tumor size (mm3)±SEM**	**Av. Final tumor size (mm3)±SEM**	**Change in tumor size (%)**	**Undetected tumors (%)**	**Death (%)**	**Av. Final tumor Weight of (mg)±SEM**
Treatment	461 ± 32.80	140 ± 49.81	−70	43	0	123 ± 45.96
Control	463 ± 48.44	750 ± 143.5	72	0	14	532 ± 103.13

#### Evaluation of Liver and Kidney Functions

Liver enzymes serum levels were evaluated for the treated and control groups; also, for the normal mice that did not have any tumors (as a reference for normal liver function). The treated group exhibited insignificant (*p* > 0.05) differences and within the range in serum ALT and AST levels compared to the normal untreated mice. Then again, the treated group showed insignificant (*p* > 0.05) differences in mean serum creatinine levels, which were lower but within the normal range compared to the normal untreated mice (Table 5).

**Table 5:** Serum ALT, AST levels (IU/L) Serum creatinine levels in (mg/dL) for different groups (treatment, negative control treated with the only vehicle, and normal untreated mice).

**Groups**	**AST (IU/L) ±SEM**	**ALT (IU/L) ±SEM**	**Creatinine (mg/dL) ±SEM**
Treatment	34 ± 0.12^*^	42 ± 0.96^*^	0.25 ± 0.008^*^
Control	56 ± 0.23	98 ± 0.31	1.10 ± 0.02
Normal	36 ± 0.55	63 ± 1.9	0.80 ± 0.05

* *P > 0.05 (statistically insignificant)*.

### Antimicrobial Assay

Ethanol extract was more effective against all tested bacteria strains and yeast than the aqueous extract. *S. aureus* was highly affected by ethanol extract with MICs of 6.25 mg/ml compared to the aqueous extract, which was 50 mg/ml. *E. coli, B. subtilis*, and *P. aeruginosa* had the same MICs for the aqueous and ethanol extracts (50 mg/ml and 25 mg/ml, respectively), while *C. albicans* was affected by both extracts with the same MICs of 25 mg/ml Table 6.

**Table 6:** Minimum inhibitory concentration (MIC) in mg/ml of Gaz alafi extracts.

**The tested microorganism**	**Gaz alafi extracts**		**Positive control**	
	**Aqueous**	**Ethanol**	Gentamicin*& Amphotericin B **
*E. coli*	50 ± 0.17	25 ± 0.17	0.11 ± 0.02
*B. subtilis*	50 ± 0.04	25 ± 0.05	0.01 ± 0.03
*P. aeruginosa*	50 ± 0.08	25 ± 0.13	0.062 ± 0.008
*C. albicans*	25 ± 0.10	25 ± 0.16	0.76 ± 0.007
*S. aureus*	50 ± 0.15	6.25 ± 0.02	0.15 ± 0.06

*The results were represented as mean ± SEM, (n = 3)*.

From the literature: *MIC value for gentamicin against S. aureus is 0.0125 mg/ml and 0.00625 mg/ml against E. coli (35). **MIC value for amphotericin B, ranging from 0.0005 to 0.002 mg/ml (36).

## Discussion

Gaz-Alafi (Manna) is a byproduct of insect activities on host plants, locally collected to use as candy or herbal therapy. This material is called Man-alsma in Arabic culture. Although this material or, maybe, similar has a special position among the other foods since it is mentioned in the three holy books, Taura, Bible, and Quran, the awareness of this food is very rare. The term “Manna” might include all the plant secretions. The specific origin of the manna extracted in the current study as a firm mass of *Q. brantii* leaves with manna exudate is the Penjwen district of Sulaymaniyah (north of Iraq) where the leaves are collected and boiled in water and then mixed with eggs to make a popular dessert (37). To the best of our knowledge, the present study is the first to evaluate Gaz-Alafi throughout immunomodulatory and antiproliferative evaluation *in vitro* and *in vivo* using water and ethanol extracts.

Phytochemical compounds give essential information about plant physiology and biochemical pathways. Middle East medicinal plants are considered attractive sources for these agents as they are widely used in traditional medicine (38, 39). The secondary metabolites in these plants are promising sources of pharmacologically active agents (40). Secondary metabolites are structurally diverse chemical compounds that are produced by plants or other living organisms. They have unusual and varied chemical structures and form a heterogeneous collection of biologically active molecules with multiple modes of action.

In general, plants' secondary metabolites, especially those obtained from edible plants and plants used traditionally, are relatively safer than synthetic products because most of them are biodegradable and have no severe reported side effects (40). Among plant-derived bioactive molecules, polyphenols are a large and heterogeneous set of secondary metabolites that include stilbenes, flavonoids, lignans, benzoic acid derivatives, and cinnamic acids, which have at least one hydroxylated aromatic ring in their structure (41). Polyphenol compounds are used as a basis of many current pharmaceuticals (42). The consumption of specific types of food rich in polyphenols is positively associated with health. For example, many edible plants showed activities as antioxidant, anti-inflammatory, immunomodulatory, and anticancer (43, 44).

Previous studies have reported the occurrence of secondary metabolites, such as glycosides, terpenoids, phenolic compounds, fatty acids, sterols, and tannins in the *Quercus* species (45). Phenolic acids, such as gallic, chlorogenic, ρ-hydroxybenzoic, vanillic, syringic, caffeic, ρ-coumaric- acids, and flavonoids, such as rutin, quercetin, naringenin, hesperetin, and kaempferol, are common in these species (39, 46, 47). Also, tannins are considered to be one of the major secondary metabolites found in the galls of *Quercus species*, such as flavan-3-ol monomers, catechins, epicatechins, gallocatechin, and epigallocatechin, alongside with gallic acid, syringic acid, ellagic acid, β-sitosterol, amentoflavone, hexamethyl ether, isocryptomerin, methyl betulate, and hexagalloyl glucose, and their derivatives (14, 48).

In the *in vitro* antiproliferative assay, decent activity on breast cancer was reported without toxic effect on fibroblast cells. The ethanol extract showed the most effective, inhibiting cancer cells at concentrations as low as 15 mg/ml, mainly on T47D and EMT-6 cells. Nevertheless, the aqueous extract was also efficient against some cell lines at varied concentrations.

Higher phenolic contents could explain the observed antiproliferative activity for ethanol extract than the aqueous extract resulted in the TPC assay, mainly gallic acid (GA) presence in both extracts. In general, the genus Quercus has high potency against inflammation and proliferation of cancer cells. The main antiproliferative compounds identified in the *Quercus* species are gallic acid, ellagic acid, kaempferol, quercetin, and myricetin (48, 49). These compounds are polyphenols that are quite efficient antiproliferative and cytotoxic agents against some types of cancer cells, with no significant toxicity toward healthy cells (50). They possess high antioxidant activity by donating electrons to the ROS to stabilize them and inhibiting enzymes that form free oxygen and nitrogen radicals, such as NOS, peroxidase, lipooxygenase, and xanthine oxidase so they can reduce the risk of developing cancer (51). A study in Iran evaluated the gallic acid (from 5 to 200 μg/ml) *in vitro* antiproliferative activity against MCF-7 cells. The IC_50_ for gallic acid was 18.5 μg/ml compared with tamoxifen, which has an IC_50_ of 19.5 μg/ml. This study showed that MCF-7 cells could be treated with GA by several effects associated with cell death, such as morphological changes and decreasing cancerous cells. In addition to that, this compound had considerable effects on the expression of apoptosis genes, such as P53, P21, and Mcl-1 (52).

On the other hand, gallic acid can directly increase the expression of p53, which is also known as TP53. P53 is a protein that functions as a tumor suppressor and has a role in the initiation of the apoptosis process (53). GA can cause overexpression in P53 in different types of cancers, such as breast cancer, which enhances cell apoptosis and leads to G0/G1 arrest in the cell cycle without affecting normal cells (52). A recent study has been done by Wang and Bao (54) to evaluate GA effect on lung cancer-bearing mice. The results showed the average volume and weight of tumors in the mice were reduced by treatment with GA. Also, the viability of lung cancer cells was decreased, and the apoptotic rate was increased in a dose-dependent behavior. GA also has a potential effect on increasing the level of antioxidant enzymes, which serve as the primary line of defense against injuries caused by free radicals, so GA can be used to treat tumors initiated from oxidative stress (55).

Among the detected compounds is Catechin (flavanols), which could have played a role in the anti-proliferation response. Catechin has an antioxidant ability, which enhances the cytotoxic effect of plant extract through scavenging ROS, chelating metal ions, affecting antioxidant and pro-oxidant enzymes balance as the similarity between catechins, and ATP in the structures could result in competitive binding to the enzymes' ATP-binding sites (56, 57). Catechin was found to inhibit the growth of cancer cells (58). Catechin has been shown to inhibit tumor growth, carcinogenesis, tumor angiogenesis, and cancer cell invasion by suppressing the induction of proangiogenic factors (59, 60). Catechin in the green tea inhibited tumor growth and suppressed specific mouse mammary carcinoma 4T1 cells (61). Also, it reduces tumor angiogenesis in estrogen receptor-negative breast cancer (62). The studies suggested that the cytotoxic effects on the cancer cells result from the antioxidant properties (63). Also, catechins induced apoptosis, cell-cycle arrest, inhibited NF-κB, and cyclooxygenase-2 (COX) overexpression *in vitro*, and animal models play a significant role in preventing cancer (64, 65). Catechin could modulate apoptosis by altering the expression of the apoptotic-related genes (66–68), such as inducing the bcl-xL and bcl-2 expression, reducing Bax expression (69–71), or upregulation of the caspases-3 and caspases-10, Fas, NF-κB p105, and p53 (72, 73).

Tiliroside is a glycosyl-oxyflavone derivative of kaempferol, which was detected in the ethanol extract using LC-MS. This compound is considered uncommon due to the small amount obtained naturally (74, 75). Tiliroside demonstrates antioxidant capability due to its scavenging activity, ability to inhibit xanthine oxidase, and increase the SOD level (76). Ideally, natural compounds with antioxidant activity could be used as alternatives to synthetic cancer therapy (77). Tiliroside is a compound with an antioxidant activity that originates at the level of IGF-IR and HIF-1 alpha-signaling [76]. It cannot be considered an effective agent for cancer therapy. Studies reported that tiliroside was inactive on several cancer cell lines (78). On the other hand, per-acetylated tiliroside (a derivative of tiliroside) was significantly more potent as a specific antiproliferative agent than many other tested compounds (such as quercetin, kaempferol, and rutin), including tiliroside itself (78).

This study assessed the innate and acquired immune response alteration caused by the Gaz alafi extracts. In the lymphocyte proliferation assay, both extracts enhanced the proliferation in the presence and absence of LPS and Con-A. The aqueous extract showed slightly higher response in comparison with the ethanolic extract. Previous studies on cancer patients have revealed higher T helper-2-acquired immune response (79), where increasing T helper-1 immune response could be resulted in anticancer immune reaction (80). IL-4 levels are associated with higher Th2 response, while, for Th1, IL-2 and INF-γ are responsible for the activation (24). In contrast, healthy people had an equilibrium between both Th1/Th2 cytokines. The current study demonstrated that aqueous extract could increase IL-2 and INF-γ levels. Besides, as well, ethanol extract was responsible for increased IL-2 levels, which could indicate a shift toward anticancer immune response (Th1 antitumor response). On the other hand, macrophage phagocytosis and pinocytosis, which are important for antitumor immune response initiating by presenting the antigen to activate the T-lymphocytes (81), both were found increased after both extract supplementations. Aqueous extract showed higher activity, and ethanol extract showed some promising results in both assays in comparison with the control.

The existing active compounds could be responsible for the immune-stimulating properties of Gaz alafi. Shruthi et al. (82) established the immunomodulating effects of gallic acid against cyclophosphamide and cisplatin-induced immunosuppression in Swiss albino mice. The study showed that gallic acid at a 100-mg/kg dose counteracted the immune suppression induced by these two anticancer drugs. This effect was due to an increase in the proliferation of total leucocyte and lymphocyte counts, leading to an increase in the immune response of the host. Gallic acid can also stimulate the innate immune response by dose dependently enhancing the phagocytes and lymphocytes. It can be used as an adjuvant with immunosuppressive drugs to reduce their side effects on the immune system (82). Lymphocyte-proliferating effect could also be due to catechin, as several studies established the catechin immunomodulatory activity by acting on cellular and humoral levels (83, 84). On the other hand, the investigations have demonstrated that tiliroside can considerably inhibit the NO, TNF-α, and IL-12 production (85). Also, researchers have found that tiliroside could significantly inhibit the production of TNF-α, iNOS, IL-8, and IL-6 (86–88).

This study revealed that the ethanol extract of Gaz alafi, which was used to treat mice implanted with breast cancer caused a significant reduction in tumor size (70%) and several undetected tumors (43%). These outcomes could be a consequence of effective anticancer agents' existence, such as gallic acid and catechin. These agents could synergistically inhibit cancerous cells proliferation *via* immune system or apoptosis stimulation. Generally, this study revealed promising *in vitro* and *in vivo* antiproliferation properties from Gaz alafi extracts, particularly through their identified phytochemicals, which had numerous antitumor mechanisms, including cell proliferation inhibition, apoptosis induction, and regulation of acquired and innate immune responses.

The ethanol extract of Gaz alafi revealed considerable antibacterial activity against Gram-positive and Gram-negative bacteria (Table 3). The extracts and selected antibiotic (Gentamicin) showed varying inhibitory activities on various bacterial strains, with a general trend that Gram-negative bacteria were more resistant than Gram-positive strains. Based on the minimum inhibitory concentration (MIC) data, the ethanol extract activity was highest (6.25 mg/ml) against *S. aureus*. In contrast, the activity against other bacteria *E. coli, B. subtilis*, and *P. aeruginosa* had almost the same MIC value (25 mg/ml). MICs for the aqueous extract were almost the same (50 mg/ml) against all assessed bacteria. Moreover, the antifungal activity of Gaz alafi extracts against *C. albicans* showed similar MIC values of (25 mg/ml) for both tested extracts. In contrast, Amphotericin B (an antifungal agent) displayed an MIC value of (0.76 ± 0.007).

Limited data are available to report the antimicrobial activity of Gaz alafi. In a previous study, Nebigil assessed *Q. branti* L. different seed extracts and fractions against four bacterial strains, including *E. coli* and *S. aureus*. Minimum inhibitory concentrations for total extract and water/methanol fraction were found to be 2.5 mg/ml, 3 mg/ml for *E. coli*, and 1.25 and 3 mg/ml for *S. aureus* (89). Another study suggested that the gall extract of *Q. brantii* L. probably acts through its anti-inflammatory and antimicrobial properties to affect colitis's biochemical and pathological parameters (14).

The observed antibacterial activity in the current study could be attributed to the activity of the major compounds (gallic acid, catechin, tiliroside), either singly or synergistically. Tannins have been traditionally used as antimicrobial and antiseptic agents. Their mode of action may depend on the inactivation of microbial adhesins, enzymes, and cell envelope transport proteins so that tannins can be toxic to bacteria, fungi, and yeasts. Quercus's tannic and gallic acids are responsible for this antimicrobial activity (90, 91). Gallic acid is produced through the hydrolytic breakdown of tannic acid using a glycoprotein esterase, namely tannase (92). A study was done by Lima et al. (93) to evaluate the effect of GA on enhancing the activity of antibiotics. The results showed that GA has a synergistic effect, with two types of antibiotics against *S. aureus*. GA reduced the MIC from 156.3 to 49.21 μg/ml when associated with Norfloxacin and from 49.21 to 2.44 μg/ml when associated with Gentamicin against *S.aureus*. Oppositely, when GA was tested against *P. aeruginosa*, results revealed insignificant MIC value. GAs antimicrobial activity depends on several mechanisms, including the inhibition of extracellular microbial enzymes required for microbial growth, direct action on microbial metabolism, and an anti-adhesion mechanism. These findings could explain the high effect of Gaz alafi against *S. aureus*.

On the other hand, the catechins exhibit only modest antibacterial activity. However, extensive chemical changes to the structure could significantly enhance antibacterial effectiveness and stability *in vivo*. Nevertheless, naturally occurring catechins have a range of activities on bacteria, other than the ability to relate to bactericidal or bacteriostatic effects, such as adjusting antibiotic sensitivity and altering the factors that control bacterial virulency expression (94). Furthermore, tiliroside did not exert remarkable activity against most pathogenic bacteria or fungi, such as various strains of *Staphylococcus aureus, P. aeruginosa, C. albicans* (95, 96). Although it has poor bacteriostatic and bactericidal activity, tiliroside could find a place in the bacterial infection's treatment. Notable research was also performed on strain SA-1199B of *S. aureus*, and concluded that adding 64 μg/ml of tiliroside decreased the MIC of the tested antibiotics by (2–128) times (97). Also, computational studies observations suggested that tiliroside can probably inhibit penicillin-binding proteins 2a (PBP2a) and 4 (PBP4), produced by methicillin-resistant *S. aureus* (98).

## Conclusion

For the first time, Gaz alafi obtained from *Q. brantii* dry leaves revealed functional food benefits, involving promising anticancer and immunomodulatory properties fulfilled in the present study. Boosting the immunity responses *via* altering innate and acquired immune systems toward the cancer suppression and the existence of biologically active components like gallic acid, which has several antiproliferation property, was indicated by the present study. One limitation of this study is the low number of detected compounds in the extract. Further analysis using additional reference standards and pure compounds isolation with NMR evaluation could result in total composition identification in the future.

## Data Availability Statement

The original contributions presented in the study are included in the article/Supplementary Material, further inquiries can be directed to the corresponding author.

## Ethics Statement

The animal study was reviewed and approved by the Research and Ethical Committee at Applied Science Private University.

## Author Contributions

All authors listed have made a substantial, direct, and intellectual contribution to the work and approved it for publication.

## Conflict of Interest

The authors declare that the research was conducted in the absence of any commercial or financial relationships that could be construed as a potential conflict of interest.

## Publisher's Note

All claims expressed in this article are solely those of the authors and do not necessarily represent those of their affiliated organizations, or those of the publisher, the editors and the reviewers. Any product that may be evaluated in this article, or claim that may be made by its manufacturer, is not guaranteed or endorsed by the publisher.
