# Supplementary material for: Gaz Alafi: A Traditional Dessert in the Middle East With Anticancer, Immunomodulatory, and Antimicrobial Activities
Source: Front Nutr. 2022 Jul 1;9:900506. doi: 10.3389/fnut.2022.900506 (PMC9283951; doi:10.3389/fnut.2022.900506)
Supplement: Supplementary file 1 [file Data_Sheet_1.docx]

Supplementary Material

# Supplementary Data

#
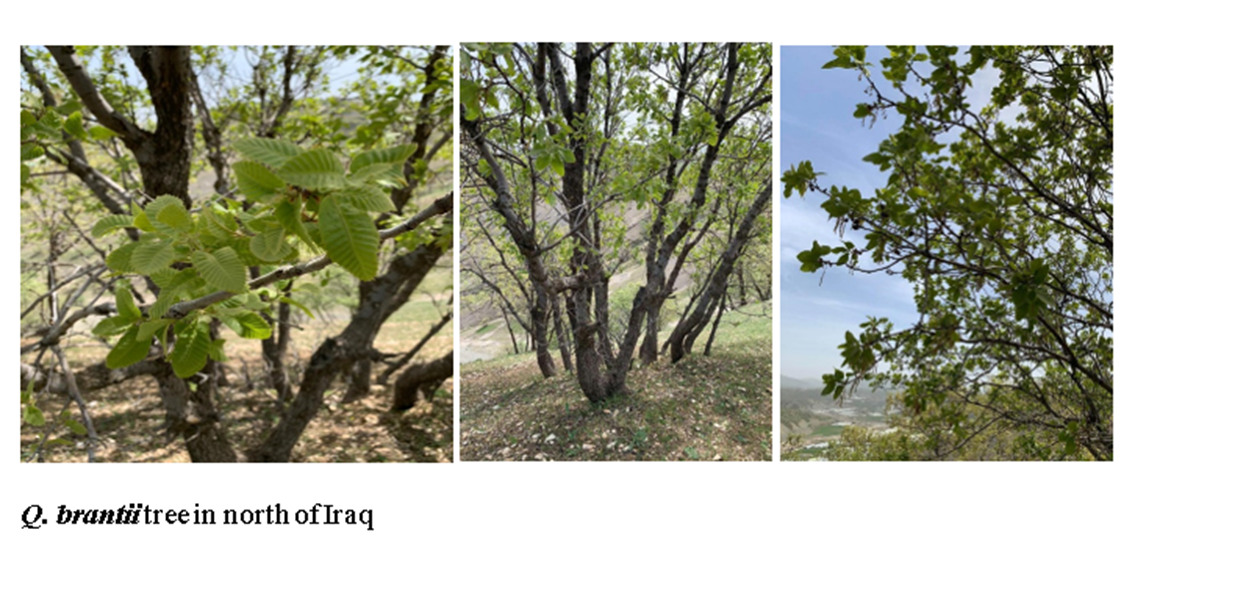

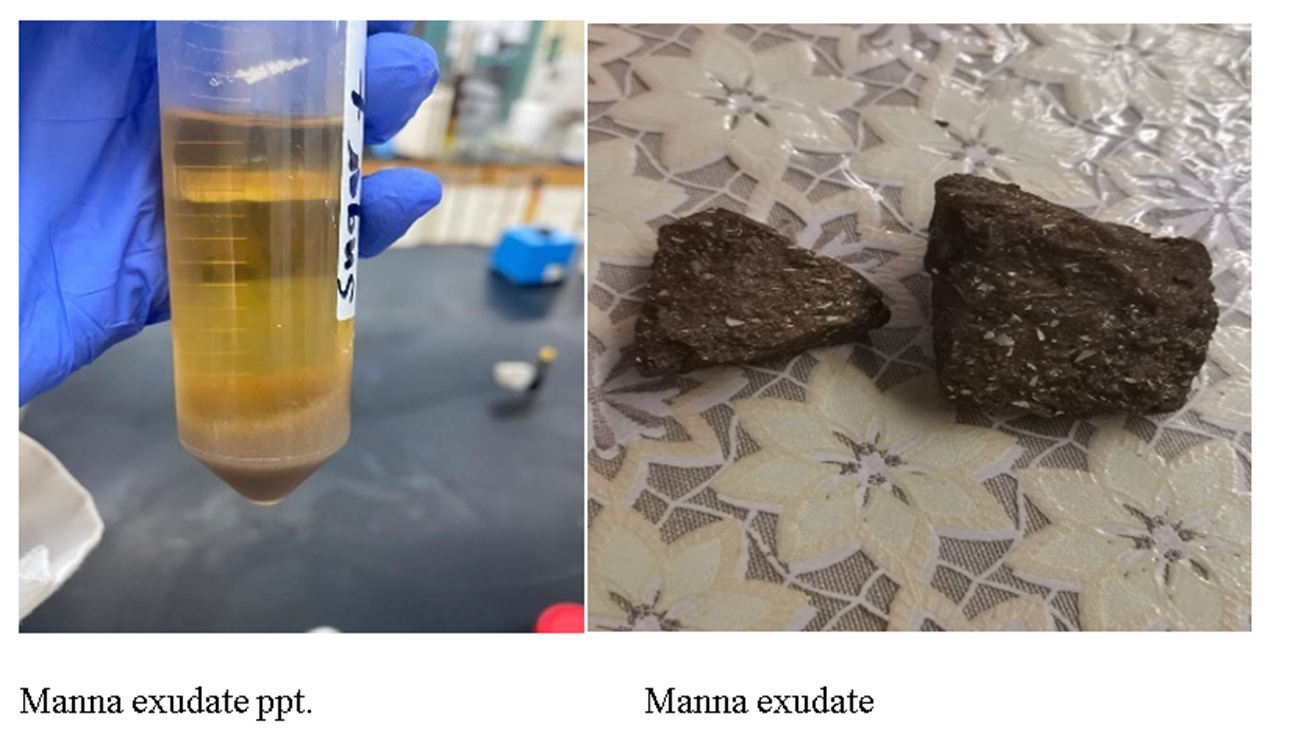


# Supplementary Figure 1. Images of the manna and the plant

**
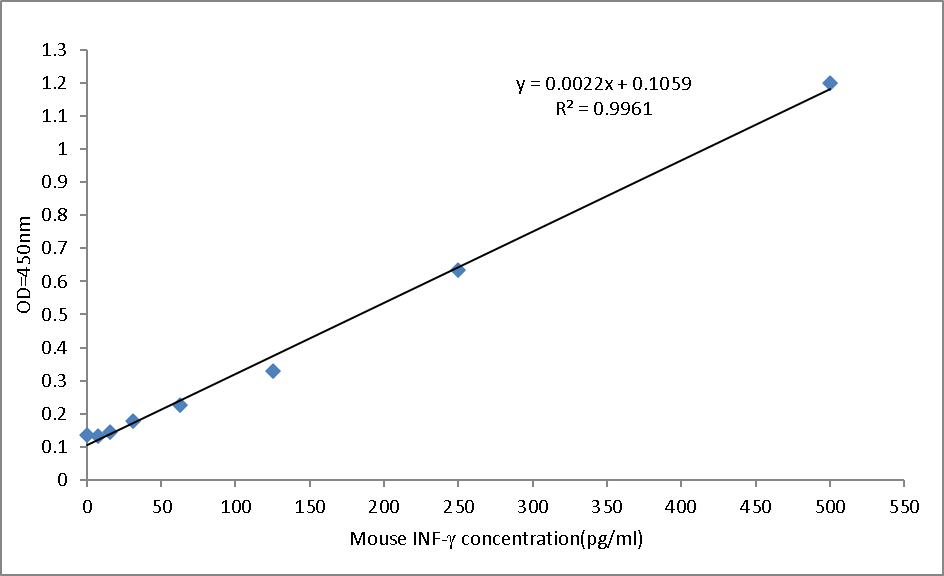
**

**Supplementary Figure 2. Standard curve for mouse INF-γ.**

**
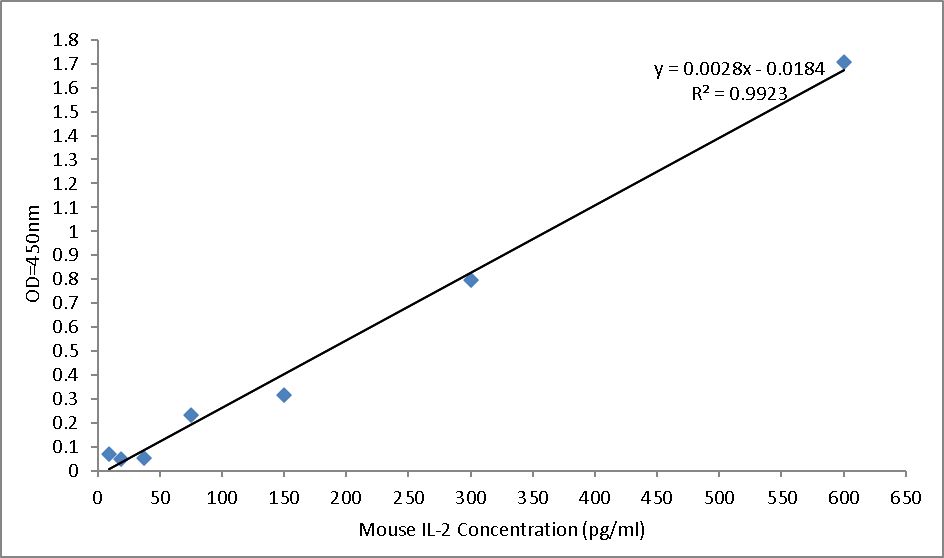
** **Supplementary Figure 3. Standard curve for mouse IL-2.**

**
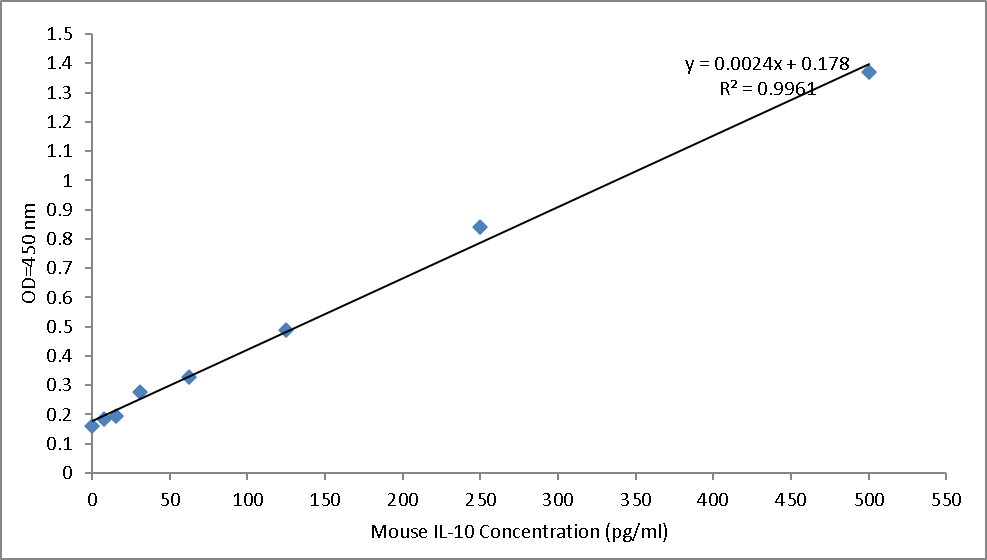
**

**Supplementary Figure 4. Standard curve for mouse IL-10.**

**
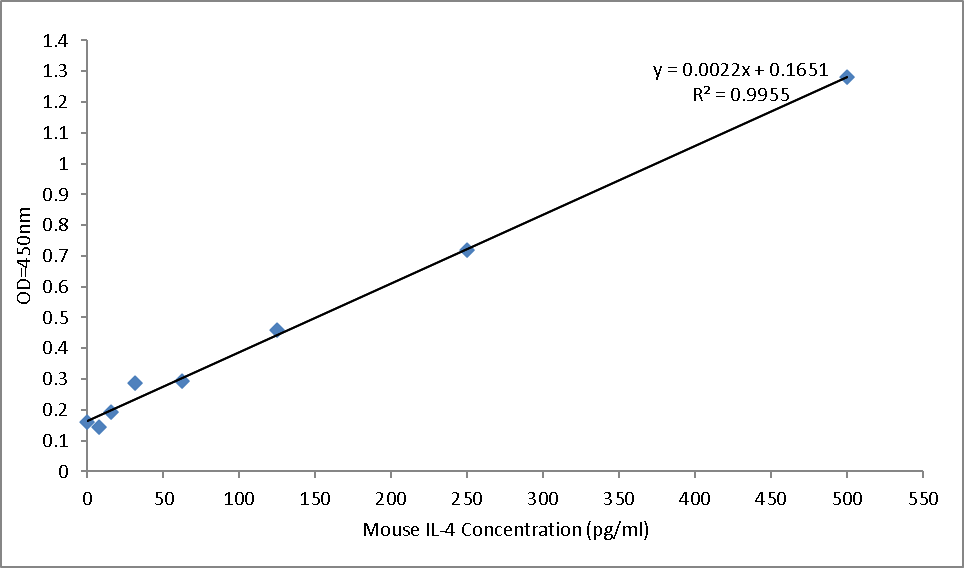
**

**Supplementary Figure 5. Standard curve for mouse IL-4.**

**
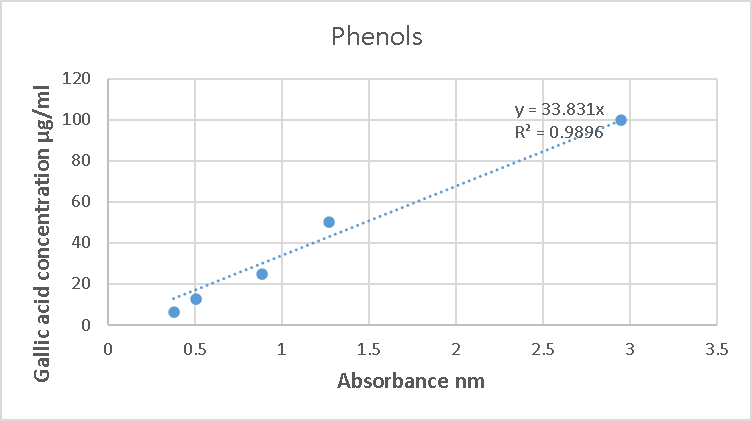
**

**Supplementary Figure 6. Standard curve for phenols.**

**
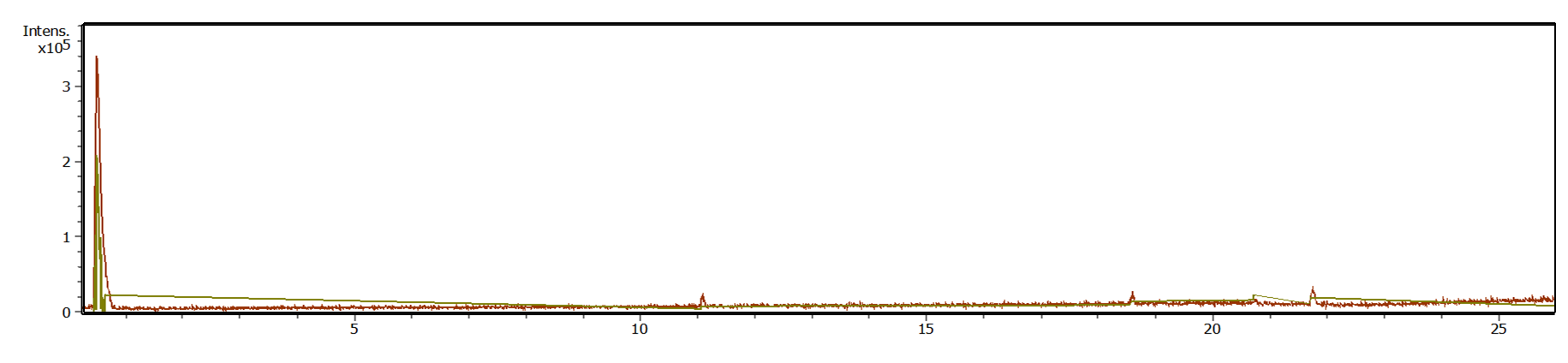
**

**Supplementary Figure 7. Ethanol extract LC-MS results**

**
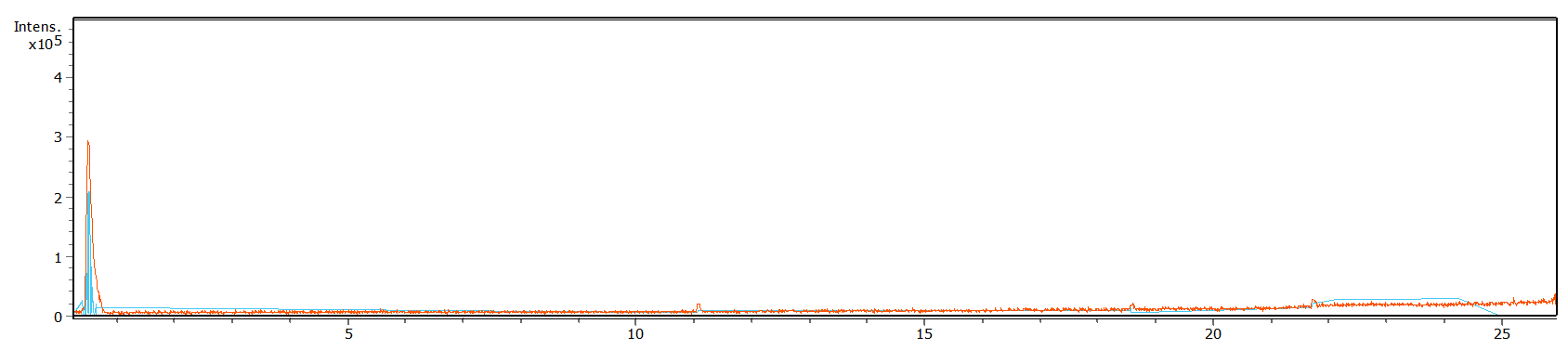
**

**Supplementary Figure 8. Water extract LC-MS results**

**Supplementary Figure 9. LC-MS standards used for this study**
